# Increasing our ability to predict contemporary evolution

**DOI:** 10.1038/s41467-020-19437-x

**Published:** 2020-11-05

**Authors:** Patrik Nosil, Samuel M. Flaxman, Jeffrey L. Feder, Zachariah Gompert

**Affiliations:** 1 Centre d’Ecologie Fonctionelle et Evolutive, Centre National de la Recherche Scientifique, Montpellier, 34293 France; 2 Department of Biology, Utah State University, Logan, UT 84322 USA; 3 EBIO, University of Colorado Boulder, Boulder, CO 80309 USA; 4 Department of Biological Sciences, University of Notre Dame, Notre Dame, IN 46556 USA

**Keywords:** Evolutionary ecology, Evolutionary theory

## Abstract

Classic debates concerning the extent to which scientists can predict evolution have gained new urgency as environmental changes force species to adapt or risk extinction. We highlight how our ability to predict evolution can be constrained by data limitations that cause poor understanding of deterministic natural selection. We then emphasize how such data limits can be reduced with feasible empirical effort involving a combination of approaches.

## What is predictability and why does it matter?

**Fig. 1:**
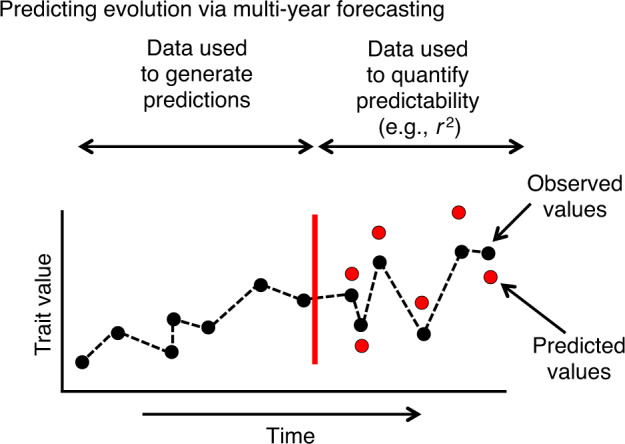
Quantifying the predictability of short-term evolution using time-series data. Autoregressive moving average (ARMA) models can be applied to existing data to generate predictions for future trait values or allele frequencies. In turn, the fit (e.g., *r*^2^ value) of these predicted values to those actually observed provides a metric of the predictability of evolution.

Prediction is a core component of the sciences. However, evolutionary biology is often portrayed as a descriptive or historical science, rather than a predictive one^1–3^. Nonetheless, the predictability of evolution can be quantified, for example by testing how well existing time series predict future evolutionary changes (Fig. 1)^1,4^. Besides its scientific importance, our ability to predict evolution has applied implications, for example for the development of vaccines and antibiotics (i.e., viruses and bacteria evolve to be resistant), animal breeding programs aimed at conservation and reintroduction, and biocontrol of insect pests that attack crops and lumber.

Here we focus on predictability defined as the ability to forecast future trait values or allele frequencies using existing data (Fig. 1). Such predictive ability can be studied using temporal data alone, or by adding information on the mechanisms and genomic basis of evolution^5^. We focus on contemporary evolution using time-series data spanning several to dozens of generations (i.e., in many organisms this will equate to decades), where evolution may proceed via standing genetic variation or new mutations. This focus on medium-term evolution complements quantitative genetics work on the predictability of immediate, single-generation responses to selection and studies that consider parallel and repeated evolution over longer (e.g., phylogenetic) time scales^3,6^.

## Two hypotheses for limits in our ability to predict evolution

**Fig. 2:**
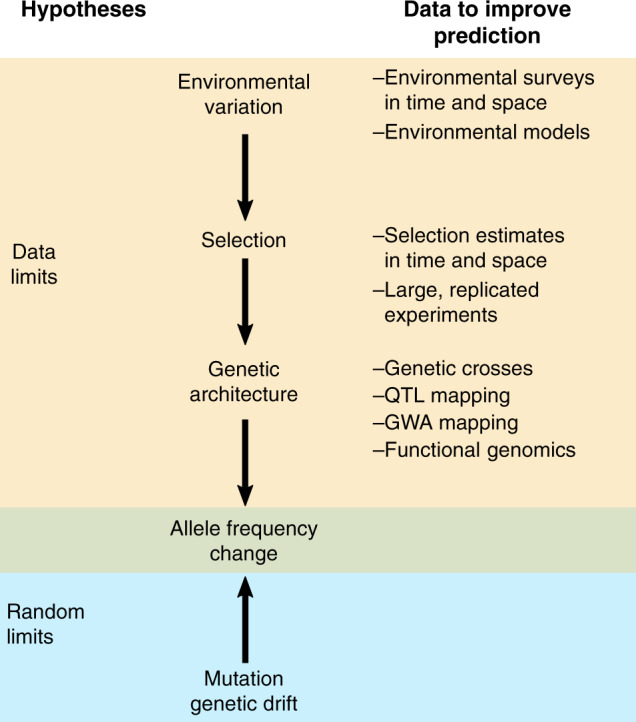
Schematic illustration of two hypotheses for limitations on predicting evolution. This includes depiction of the evolutionary processes involved, and data which might be used to improve prediction. QTL quantitative trait locus, GWA genome wide association.

The degree to which evolution is predictable forms a long-standing debate in biology^3,7^. At the core of this debate is the question of the extent to which evolution is driven by random versus deterministic processes^3^ (Fig. 2). In this context, there are two main classes of explanation for difficulties in predicting evolution. First, predictability can be limited by random processes (the “random limits” hypothesis)^8^. The key mechanisms underlying this hypothesis are stochastic changes in allele frequency due to genetic drift and the random nature of mutation. Second, even evolution driven by deterministic natural selection can be difficult to predict, due to limited data that in turn leads to poor understanding of selection and its environmental causes, trait variation, and inheritance^4,9,10^ (the “data limits” hypothesis). Indeed, a starting point for improving our ability to predict evolution is to increase understanding of when selection is expected to be directional, fluctuating, or stabilizing.

These explanations are not mutually exclusive and are likely to operate simultaneously. However, they are conceptually distinct due to core differences in the factors that they propose to limit our ability to make accurate predictions; inherent unpredictability caused by stochastic processes underlies the random limits hypothesis, whereas insufficient knowledge on the part of those trying to form predictions underlies the data limits hypothesis.

In terms of the data limits hypothesis, the underlying assumption is that with sufficient data and proper analysis, deterministic processes can be predicted. Thus, shortcomings in predictive ability stem largely from insufficient data and inadequate analytical tools, not from inherent randomness per se. Limits to data and our understanding of evolutionary process can arise at several levels. First, the environmental sources of selection, such as climatic conditions or predator abundance, might fluctuate in ways that are themselves difficult to predict, even if they are deterministic^1^. We stress that even deterministic environmental fluctuations might appear random, due to sensitivity to initial conditions that generates chaotic dynamics^11^. Such chaotic fluctuations are not truly random and our ability to predict them is still, in principle, tied to data limits. Second, even if environmental changes can be predicted, poor understanding of how environmental factors affect resource distributions and impose selection on phenotypes can reduce predictive ability for trait evolution. Third, poor understanding of the genetic architecture of traits can produce difficulties predicting genetic change from patterns of phenotypic selection^5,12^. For example, prediction can be complicated by phenotypic plasticity, which may be a common way that organisms respond to environmental change^13^.

At all these levels, limits can arise in the quality or quantity of data, and in analysis. Such data limits are exacerbated by the potential for different factors to act at varying temporal and spatial scales, and by the fact that rare and difficult to predict environmental changes can have large effects on evolution. These general concepts apply across environmental factors, traits, and taxa, as outlined in Box1 using examples in birds, insects, and other organisms.

**Fig. 3:**
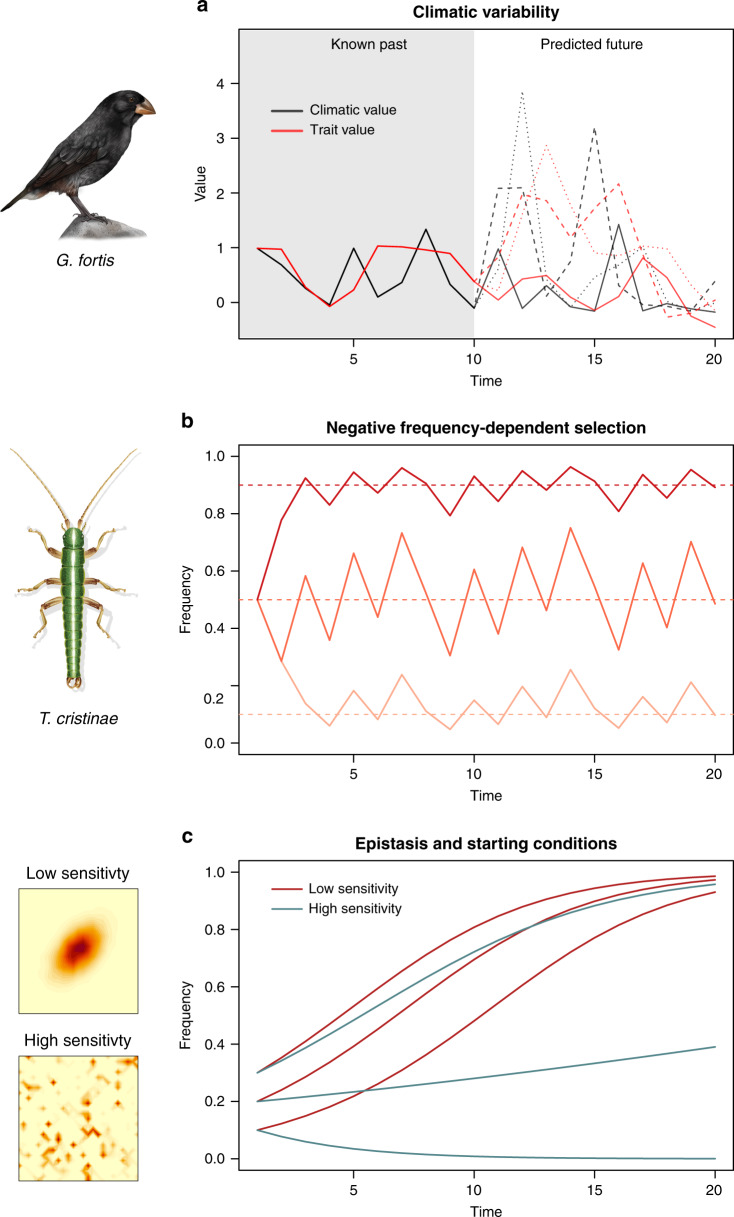
Hypothetical examples of how variation in different factors can limit the predictability of evolution driven by deterministic natural selection. This figure is motivated by empirical systems, but does not depict real data. **a** Uncertainty in climatic variability can limit the predictability of evolution for traits affected by environment-dependent fluctuating selection, such as beak size in *G. fortis*. Here black lines denote observed (left half) or predicted (right half) climatic values, and red lines denote observed (left half) or predicted (right half) trait values. Multiple possible predictions are shown. **b** Uncertainty in the form of the selection function can limit the predictability of evolution by negative frequency-dependent selection, as is observed for color pattern in *T. cristinae* stick insects. Possible evolutionary trajectories given three different selection functions (different colored lines) are shown here. **c** Predictability can also be limited by sensitivity to initial conditions, as occurs on rugged fitness landscapes with considerable epistasis. Two hypothetical fitness landscapes with low (top) and high (bottom) epistasis, and thus sensitivity to initial conditions, are shown (left side; the axes represent genotypes for different loci). Hypothetical evolutionary trajectories from different starting conditions are shown on the right (colored lines). High epistasis promotes different outcomes dependent on initial conditions. Finch and stick insect drawings courtesy of R. Ribas.

Box 1. Examples of our ability to predict evolution in natural populations We here discuss progress and challenges in predicting evolution using empirical examples. A first example involves fluctuating selection caused by climatic variability, which has been documented in numerous species^26–28^; (Fig. 3a). Perhaps the best example stems from long-term studies of beak size evolution in Darwin’s finches^1^. Here, variation in rainfall on Daphne Major has been shown to affect the relative abundances of small versus large seeds, which in turn can exert selection on beak size in *Geospiza fortis* during drought conditions. Thus, rare and difficult-to-predict droughts can have large effects on evolution. Indeed, in the case of *G. fortis* it could be argued that evolution is unpredictable not because we don’t understand selection (i.e., selection is known to be exerted by seed size distributions), but rather because available data and models cannot predict climatic fluctuations, or how these affect seed size distributions. Thus, prediction in this case was limited (*r*^2^ ~ 0.14, this value is a point estimate from autocorrelation analysis of how well trait values for beaks in the past predict those in the future, see also Fig. 1)^4^, and might be improved via better climate models and data on how climate affects resource distributions.

A second example involves predation, which is a common source of natural selection that can fluctuate according to prey characteristics (Fig. 3b). In particular, predation can cause negative frequency-dependent selection (NFDS) when predators focus on more common prey types. In such cases, the fitness of a phenotype fluctuates because it depends on the phenotype’s frequency in the population, and is higher when the phenotype is rare. This has been documented, for example, in cichlids, guppies, stickleback, and stick insects^4,29–31^. Such systems represent cases where evolution is expected to be easier to predict. Even with NFDS, however, data limits can apply, as illustrated by long-term studies of the evolution of striped and unstriped cryptic color morphs in *Timema cristinae* stick insects^4^. In *T. cristinae*, morph frequencies fluctuate predictably among years (*r*^2^ ~ 0.90) and there is experimental support for NFDS. Specifically, an experiment showed that the striped morph is strongly favored when initially rare (i.e., 20% initial frequency), but shows idiosyncratic changes when initially common (80% initial frequency). Whether selection would differ if ratios were manipulated more extremely is unclear. Moreover, why fluctuations occur at yearly, rather than monthly, scales is unknown. Thus, prediction might be improved by estimating the quantitative form of the NFDS fitness function, and via understanding factors that affect the foraging behavior and search images of bird predators. Nonetheless, evolution was highly predictable in this example, and the mechanisms of evolution are reasonably understood due to insights from combining experiments and genomics. Specifically, experiments support NFDS and genomic data rule out a predominant role for random genetic drift, and have clarified the role of epistasis^32^ and suppressed recombination in the evolution of color genes.

## Challenges and ways forward

The examples in Box1 illustrate how data limits in even well-studied systems can mediate the extent to which scientists can predict evolution. However, rather than dampening hope for prediction, the results suggest that progress can be made with empirical effort, for example via coupling long-term monitoring of populations with large, replicated experiments that reveal evolutionary process, and powerful genomic tools that allow dissection of the genetic basis of traits. Nonetheless, gathering such data will rarely be a trivial task. At a minimum, obtaining time-series data necessarily takes time, and this cannot be sped up with more effort. Identifying and measuring additional factors affecting evolutionary dynamics, such as relevant environmental parameters and selection estimates, increases the effort required. Simulation models calibrated based on empirical understanding of a system may aid in parsing the effects of different factors on predictability (e.g., variation in selection, genetic architecture, random drift), thus guiding researchers as to where further effort is best placed, the sample sizes required to increase precision, etc. Box 1 provides specific examples of how knowledge of a study system can inform where additional empirical effort is best placed, and Table 1 lists analytical tools that enable prediction. Thus, we propose that focused data collection and analysis can improve prediction of evolution. However, we temper this claim with the caveat that this will not necessarily be an easy task, particularly because the required measurements potentially span different scales of time, space, and biological organization.

**Table 1:** Examples of data types and models that can aid the quantification of uncertainty related to predicting evolution over moderate time scales.

Data type	Model	Key features	Software (citation)
Trait genetics	Bayesian sparse linear mixed model (BSLMM)	Estimates heritabilities, genetic covariances and number of causal genetic variants while accounting for (and quantifying) uncertainty in genotype-phenotype associations	GEMMA^12^
Climatic variation	Bayesian modeling of uncertainty in ensembles of climate models	Generates future, predictive distributions of climatic variation with uncertainty over different climate models	JAGS/STAN^21^
Ecological interactions	N-level structural equation modeling (e.g., generalized linear latent and mixed models (GLLAMM))	Multilevel extension of structural equation modeling that allows for interactions across hierarchical levels in a Bayesian context; can consider joint uncertainty of model parameters and latent variables	xxM^22^
Evolution	Forward genetic simulation models (e.g., Wright-Fisher and extensions with age structured populations, etc.)	Flexible models that allow for drift, selection, gene flow, and other evolutionary processes; can be fit in various ways, and can incorporate ecological data	SLiM3^23^
Time series	Autoregressive moving average models (ARMA)	Models that account for spatial or temporal autocorrelation; of broad and general use for time-series analysis	JAGS/STAN^24^
Combination of data types	Hierarchical (multilevel) Bayesian models	General class of flexible Bayesian models that can combine disparate types of data to make joint inference of evolutionary processes, considering uncertainty from each source and integrated over sources	JAGS/STAN^25^

We focus mostly on hierarchical (i.e., multilevel) models that can be fit in a Bayesian context. Each model accounts for uncertainty (due to data limits or randomness) in a factor relevant for predicting evolution, but an ideal analysis would combine these components to propagate information and uncertainty across these disparate components. We stress that the examples below are representative, but by no means exhaustive.

Moreover, many complexities make it difficult to obtain data sufficient for accurate prediction (Fig. 3). An example of such a complexity is where mutations interact with one another (i.e., epistasis), rather than having additive effects. Epistasis can cause some genotypic combinations to have much higher fitness than others. Thus, epistasis can cause even adaptive (i.e., non-neutral) evolution to be mediated by historical contingencies in the type and order of mutations that arise^14,15^. Specifically, mutations that arise early in evolution can strongly affect which mutations are subsequently viable, making evolution dependent on mutation-order and difficult to predict. For example, mutations that arise early in the evolution of antibiotic resistance effect which subsequent mutations are favored by natural selection^15^. Other interactions, such as those between genes and the environment, are likely to have similar effects for complicating prediction.

A related issue is sensitivity to initial conditions^11^, which can lead to chaotic dynamics that are deterministic but impossible to predict unless initial conditions are known with extreme precision. An example where this might occur is evolution on highly rugged fitness landscapes, where ruggedness arises due to epistasis. Here, the starting place on a rugged landscape might strongly affect which local fitness peaks are climbed and which valleys are difficult to cross. Although biology may not have a strict counterpart to the Heisenberg uncertainty principle, it is possible that data collection itself alters starting conditions for evolution (e.g., if a human observer scares away predators, this could affect predator-prey dynamics for subsequent evolution). Chaos has received much attention outside of the biological sciences and in the field of ecology, but is not often considered in evolution.

All this said, there are also reasons for hope. For example, conceptual and analytical frameworks from the scientific study of complex systems exist to aid prediction of complex phenomena (Table 1). Specifically, systems thinking focuses on understanding and predicting how complex networks exhibit emergent properties not shown by individual nodes in the network^16^. In terms of evolution, this involves considering the dynamics of collective networks of genes, populations, and interacting species, rather than trying to use reductionist approaches to understand components in isolation. Because systems approaches apply across scientific disciplines a qualitative analogy can be drawn between the current state of a biological population and the ability to predict its future state based on knowledge of the evolutionary forces operating on it, and the current state of a physical system and the ability to predict its future state based on knowledge of the physical forces acting upon it. In both physics and biology there is the distinction between predictions for individual particles or genes versus the aggregate behavior of many particles (as in statistical thermodynamics) or genes (leading to quantitative genetic breeding values)^17^.

## Conclusions

In conclusion, although collecting sufficient data for prediction may often represent a formidable challenge, we argue that it is not an insurmountable one. With creative application of emerging technologies and analytical approaches we may improve our ability to predict evolutionary patterns and processes. For example, genomic tools will allow the inference of genetic details such as non-linearities in the genotype-phenotype-fitness map^18^, which can then be incorporated into models to improve prediction. Box 1 provides an example where genomic tools, experiments, and knowledge of genetic and ecological interactions were used to aid prediction of evolution in stick insects. In turn, improved ability to predict evolution may affect our understanding of ecological processes, because to the extent that evolution can be predicted, perhaps so can its ecological consequences for communities and ecosystems^19^.

A major avenue for future work is to expand the concepts presented here across broader time scales, where the probability of rare yet consequential events increases. Such longer-term prediction will likely require combining contemporary time series data with deeper phylogenetic patterns, and experimental tests of evolutionary processes. Indeed, progress on this front is exemplified by long-term experimental evolution studies in microbes that demonstrate the effects of rare yet consequential random mutations^20^. Although only further work can reveal the extent to which prediction can be realistically improved, we propose that appreciable progress should be possible in at least some species.

## References

[1] Grant PR, Grant BR (2002). Unpredictable evolution in a 30-year study of Darwin’s Finches. Science.

[2] Lässig, M., Mustonen, V. & Walczak, A. M. Predicting evolution. *Nat. Ecol. Evol*. **1**, 77 (2017). 10.1038/s41559-017-0077 28812721

[3] Blount, Z. D., Lenski, R. E. & Losos, J. B. Contingency and determinism in evolution: replaying life’s tape. *Science* **362**, eaam5979 (2018). 10.1126/science.aam5979 30409860

[4] Nosil P (2018). Natural selection and the predictability of evolution in *Timema* stick insects. Science.

[5] Exposito-Alonso M, Burbano HernánA, Bossdorf O, Nielsen R, Weigel D (2019). Natural selection on the* Arabidopsis thaliana* genome in present and future climates. Nature.

[6] Stern, D. L. *Evolution, Development, & the Predictable Genome* (Roberts & Co. Publishers, USA, 2011).

[7] Reznick DN, Travis J (2018). Is evolution predictable?. Science.

[8] Gould, S. J. *The Structure of Evolutionary Theory* (Harvard University Press, USA, 2002).

[9] Reimchen TE (1995). Predator-induced cyclical changes in lateral plate frequencies of *Gasterosteus*. Behaviour.

[10] Marques DA (2018). Experimental evidence for rapid genomic adaptation to a new niche in an adaptive radiation. Nat. Ecol. Evol..

[11] Rego-Costa A, Débarre F, Chevin L-M (2018). Chaos and the (un)predictability of evolution in a changing environment. Evolution (N. Y).

[12] Zhou, X., Carbonetto, P. & Stephens, M. Polygenic modeling with bayesian sparse linear mixed models. *PLoS Genet*. **9**, e1003264 (2013). 10.1371/journal.pgen.1003264 PMC3567190 23408905

[13] Pfennig DW (2010). Phenotypic plasticity’s impacts on diversification and speciation. Trends Ecol. Evol..

[14] Storz JF (2016). Causes of molecular convergence and parallelism in protein evolution. Nat. Rev. Genet..

[15] Weinreich DM, Delaney NF, DePristo MA, Hartl DL (2006). Darwinian evolution can follow only very few mutational paths to fitter proteins. Science.

[16] Kitano H (2002). Systems biology: a brief overview. Science.

[17] de Vladar HP, Barton NH (2011). The contribution of statistical physics to evolutionary biology. Trends Ecol. Evol..

[18] Milocco L, Salazar-Ciudad I (2020). Is evolution predictable? Quantitative genetics under complex genotype-phenotype maps. Evolution.

[19] Hendry, A. P. *Eco-evolutionary Dynamics* (Princeton University Press, USA, 2017).

[20] Blount, Z.D., Borland, C.Z., & Lenski, R.E. Historical contingency and the evolution of a key innovation in an experimental population of *Escherichia coli**Proc. Natl Acad. Sci.* **105**, 7899–7906 (2008). 10.1073/pnas.0803151105 PMC2430337 18524956

[21] Tebaldi C, Knutti R (2007). The use of the multi-model ensemble in probabilistic climate projections. Philos. Trans. R. Soc. A Math. Phys. Eng. Sci..

[22] Rabe-Hesketh S, Skrondal A, Pickles A (2004). Generalized multilevel structural equation modeling. Psychometrika.

[23] Haller BC, Messer PW (2019). SLiM 3: forward genetic simulations beyond the Wright-Fisher model. Mol. Biol. Evol.

[24] Epperson, B. K. *Geographical Genetics (MPB-38)* (Princeton University Press, USA, 2003).

[25] McElreath, R. *Statistical Rethinking: A Bayesian Course with Examples in R and Stan *(CRC Press, USA, 2020).

[26] Siepielski AM, DiBattista JD, Carlson SM (2009). It’s about time: the temporal dynamics of phenotypic selection in the wild. Ecol. Lett.

[27] Siepielski AM (2017). Precipitation drives global variation in natural selection. Science.

[28] Bergland AO, Behrman EL, O’Brien KR, Schmidt PS, Petrov DA (2014). Genomic evidence of rapid and stable adaptive oscillations over seasonal time scales in *Drosophila*. PLoS Genet..

[29] Olendorf R (2006). Frequency-dependent survival in natural guppy populations. Nature.

[30] Bolnick DI, Stutz WE (2017). Frequency dependence limits divergent evolution by favouring rare immigrants over residents. Nature.

[31] Hori M (1993). Frequency-dependent natural selection in the handedness of scale-eating Cichlid fish. Science.

[32] Nosil, P. et al. Ecology shapes epistasis in a genotype-phenotype-fitness map for stick insect colour. *Nat. Ecol. Evol.* 10.1038/s41559-020-01305-y (2020). 10.1038/s41559-020-01305-y 32929238

